# Magendie’s Method of Treating Neuralgia*Medico-Chirurgical Review, July, 1841, p. 202.

**Published:** 1841-11

**Authors:** 


*Magendie's Method of treating Neuralgia.**—The remedy, *par excellence*, recommended by M. Magendie in the treatment of obstinate neuralgia of the face and other parts, is electro-acupuncture. The needles should be made of an unoxydisable metal, and therefore those of platina are to be preferred. With respect to the mode of introducing them, it is better to push them at once, and with a sort of plunge, than to endeavor to drill them more slowly. In most cases two needles are quite sufficient; one near the origin of the nerve, and the other near its termination or expansion. The former is then to be connected with the positive wire, and the latter with the negative wire of a galvanic apparatus. The patients usually describe the sensations experienced as if a spark or stream of lightning passed instantaneously along all the divisions of the nerve: at the same time the muscles of the part are thrown into contractions. The application is not to be continued beyond a few cases, except in some severe cases, in which a continued stream must be maintained for some time. M. Magendie gives the preference to Clarke's electro-magnetic machine, as being altogether more convenient than any other for the purpose of electro-acupuncture.

If the neuralgic pain leaves one branch of a nerve to fix itself upon another branch, or upon another nerve, one or both needles are to be withdrawn, and should be inserted along the course of the nerve newly affected.

Several cases of supra-orbital neuralgia are adduced, in which the employment of electro-acupuncture speedily dispelled all suffering. The following one, in which the superior maxillary branch of the fifth pair was the affected nerve, may deserve to be noticed.

---

*Medico-Chirurgical Review, July, 1841, p. 202.

M. Thelin had been subject to frequent attacks of most severe neuralgia, affecting the superior maxillary nerve of the left side, when he first consulted M. Gagendie. The pain in the gums, lips, cheek, and ala nasi, were insupportable; the patient could scarcely utter a word, and as for mastication, that was impossible. All methods of treatment had been tried, and all tried in vain. What with having many of his teeth extracted, and being leeched and blistered, and physicked for months and months at a time, his constitution had suffered severely. He consulted M. Magendie on the 5th of March, 1838; at one sitting of a few minutes the pain was *chasse.* Since that period, whenever the neuralgia returned, he repaired to M. Magendie, and always left him cured of his sufferings. It is now several months since he has had an attack.

In the second volume of our author's lectures on the nervous system, he has related two cases of severe ueuralgia affecting the tongue; in one of which the disease had lasted for four, and in the other, for one year. . . . . "A very fine platina needle was inserted into the trunk of the facial nerve, where it enters the parotid gland, and another was inserted into the affected side of the tongue. In this manner I was sure to act on the seventh and fifth pairs of nerves, since I punctured the trunk of the first and the lingual branch of the second. The needles were then connected with the wires of Clarke's machine. In one of the patients the pain in the tongue immediately ceased, but it fixed itself on the mental branch of the inferior maxillary nerve. The needle was forthwith withdrawn from the tongue, and inserted over the *foramen mentale.* The pain was driven from this point, but it was almost immediately transferred to the infra-orbital nerve. The needle was, therefore, introduced over the aperture from which it escapes. The enemy was thus pursued from one point to another, and ultimately was expelled before the patient left my house. In the other case, the pain, when driven from the tongue, took refuge in the sub-orbital nerve; driven from this, it returned to the tongue, whence it was again dislodged. Ultimately the patient was quite cured."

Certainly such practice is infinitely superior to that of attempting to divide each nerve, that becomes successively affected, as practised, for example, by M. Roux in a recent case, where he divided first the mental branch of the inferior maxillary, then the lingual, and, lastly, the sub-orbital nerve—the enemy, however, retreated to the ethmoidal, where the knife of the surgeon could not reach him.

"In such a case," says M. Magendie, "I pursue the pain, not with the bistoury, but with the galvanic current. Even should it fix itself upon the ethmoidal nerve, I should insert one needle into the nostril, and another into the orbit, along the upper part of its internal wall, at the place where the external nasal traverses it, thus attacking it both near its origin and its termination."—*Gazette Medicale.*

*Remark.*—We have no doubt that electro-acupuncture will relieve the suffering in many cases of neuralgia which are unconnected with structural disease of any part; but it is more than probable that the relief will be only temporary, unless appropriate constitutional means are employed at the same time.—*Rev.*

